# Characteristics of salivary telomere length shortening in preterm infants

**DOI:** 10.1371/journal.pone.0280184

**Published:** 2023-01-17

**Authors:** Lisa M. Schneper, Amanda J. Drake, Taylor Dunstan, Iulia Kotenko, Daniel A. Notterman, Chinthika Piyasena

**Affiliations:** 1 Department of Molecular Biology, Princeton University, Princeton, NJ, United States of America; 2 Queen’s Medical Research Institute, University of Edinburgh, Edinburgh, United Kingdom; University of Newcastle, UNITED KINGDOM

## Abstract

**Objective:**

To examine the association between gestational age, telomere length (TL) and rate of shortening in newborns.

**Study design:**

Genomic DNA was isolated from buccal samples of 39 term infants at birth and one year and 32 preterm infants at birth, term-adjusted age (40 weeks post-conception) and age one-year corrected for gestational duration. Telomere length was measured by quantitative real-time PCR. Demographic and clinical data were collected during clinic or research visits and from hospital records. Socioeconomic status was estimated using the deprivation category (DEPCAT) scores derived from the Carstairs score of the subject’s postal code.

**Results:**

At birth, preterm infants had longer telomeres than infants born at term. However, there was no difference in telomere length between preterm infants and term infants at one year of age, implying that the rate of telomere shortening was greater in pre-term than term infants. Interestingly, TL at age 40 weeks post-conception in preterm infants was significantly longer than term infant TL at birth, suggesting that time since conception is not the only factor that affects rate of shortening. Several factors, including sex, fetal growth restriction, maternal age, maternal booking body mass index (BMI), mother education level and DEPCAT score, also differed between the preterm and term groups.

**Conclusions:**

Preterm infants have longer telomeres than term infants at birth. In the studied cohort, the rate of telomere shortening was greater in the premature group compared with the term infants. This finding agrees with previous studies using cord blood, suggesting that the longer TL in premature infants detected at birth do not persist and demonstrating that use of saliva DNA is acceptable for studies of telomere dynamics in infants. However, that the TL at age 40 weeks post-conception in preterm is longer than term infants at birth suggests that biological factors other than time since conception also affect rate of shortening.

## Introduction

Preterm birth is defined by the World Health Organization as birth before 37 completed weeks of gestation [1]. Between March 2017 and March 2018 in Scotland, 8.3% of all births (6.6% live singleton births and 68% of live multiple pregnancy births) were premature in contrast to the 1970’s when approximately 5.5% (5.0% of singleton live and 32.9% of multiple live births) were premature [2]. This increase in preterm births has been attributed in part to increases in the occurrence of multiple births due to assisted reproductive techniques, non-spontaneous pre-term deliveries due to improvements in maternity and neonatal care, maternal age, and underlying maternal health issues such as diabetes and high blood pressure [2–5].

Telomeres are repetitive DNA sequences (TTAGGG repeats) located at the ends of chromosomes [6]. In most post-natal somatic cells (excluding stem cells and germ cells), telomerase is repressed so that telomere length (TL) decreases progressively with each cell division [7]. When telomeres are sufficiently short, the cell enters a state of replicative senescence and stops dividing [8–10]. This process means that generally TL of non-stem cells decreases with age [11]. Thus, the telomere has been referred to as a “mitotic clock” [12] and telomere length has been construed as a measure of “biological age” [13]. Consistent with these considerations, peripherally measured TL has been shown to be associated with a wide range of disease and health morbidities in adults [14–28] and children [29–38] and has become a popular biomarker for stress and accelerated biological aging [10, 35, 39–45]. Thus, TL at birth and the course of TL shortening in blood or saliva may provide insight into associated between premature birth and future health trajectory.

Studies of TL in preterm infants suggest that preterm infants have longer telomeres than term infants and that telomere shortening is more rapid in preterm infants than term. Friedrich *et al*. [46] observed that umbilical cord blood DNA telomere length at 32 weeks gestation was significantly shorter than that at 27 weeks. Vasu *et al*. [47] found that preterm infants (defined as < 32 weeks gestation) had significantly longer telomeres at birth and at term equivalent age than term infants in leucocytes. TL was also negatively correlated with birth weight and positively correlated with maternal age.

The current study extends these findings by measuring telomere length of buccal DNA from a cohort of preterm infants (n = 32) collected at birth, term age, and one year and term infants (n = 39) at birth and one year. We compared maternal and birth characteristics, socioeconomic and health factors, telomere length and telomere length shortening between the two groups and within groups. To our knowledge, this is the first study to compare telomere length from buccal samples in preterm and full-term infants at these time points and longitudinally.

## Results

### Characteristics of the preterm and term groups

Saliva samples were obtained for telomere length analysis from 35 preterm infants and 39 term infants. The maternal and infant characteristics differed between the two groups (Table 1). There were more males in the preterm group (*p* < 0.05) and the preterm infants had lower birth weight Z-scores indicating fetal growth restriction (*p* < 0.05). Neither paternal age nor incidence of identified maternal chronic illness differed between the term and preterm groups. The average age of the mothers of preterm infants was 31.6 years and of term infants was 35.3 years (95% confidence interval (CI) -6.3 to -1.3, *p* = 0.004). The mothers of preterm infants had a higher body mass index (BMI) > 30; *p* = 0.009). Of mothers of preterm infants, 31.4% smoked cigarettes during pregnancy compared with 5.1% of mothers of term infants (*p* = 0.004). Mothers of term infants were more likely to have attended post-secondary school (*p* < 0.001), and to live in a postal code sector ascribed a deprivation category score (DEPCAT) greater than or equal to 3 (affluent; *p* = 0.005).

**Table 1:** Descriptive statistics for infants and their parents by preterm and term birth (n = 74).

Variable	Mean ± SD, Percent [n] ^*a*^		
	Preterm	Term	Statistics
*Infants in cohort*	35	39	
*Maternal Age (years)* **	31.6 ± 5.9 [35]	35.3 ± 4.6 [39]	*t* = -3.0 (CI: -6.3 to -1.3), df = 63.82, *p* = 0.004
*Paternal Age (years)*	33.2 ± 5.6 [31]	35.3 ± 5.3 [39]	
*Maternal Body Mass Index (BMI)*	27.3 ± 7.2 [33]	24.2 ± 3.1 [39]	*p* = 0.009
Underweight (<18.5 kg/m^2^)	3.0 [1]	0.0 [0]	
Normal (18.5–24.9 5 kg/m^2^)	42.4 [14]	69.2 [27]	
Overweight (25–29.9 5 kg/m^2^)	24.2 [8]	25.6 [10]	
Obese** (> = 30 5 kg/m^2^)	30.3 [10]	5.1 [2]	
*Maternal Smoking* **^b^			
Never-smoker	42.9 [15]	64.1 [25]	*p* = 0.004
Former smoker pre-pregnancy	25.7 [9]	30.8 [12]	
Former smoker during pregnancy	11.4 [4]	5.1 [2]	
Current smoker	20.0 [7]	0.0 [0]	
Maternal Education*** ^*c*^			
Primary	5.7 [2]	0.0 [0]	*p* < 0.001
Secondary	77.1 [27]	28.2 [11]	
Tertiary	17.1 [6]	66.7 [26]	
Post-graduate	0.0 [0]	5.1 [2]	
*DEPCAT* ** ^*d*^			
1	5.7 [2]	25.6 [10]	*p* = 0.005
2	11.4 [4]	17.9 [7]	
3	20.0 [7]	28.2 [11]	
4	45.7 [16]	15.4 [6]	
5	11.4 [4]	10.3 [4]	
6	5.7 [2]	0.0 [0]	
7	0 [0]	2.6 [1]	
*Maternal Chronic Illness*			
Yes	35.3 [12]	27.8 [10]	
No	64.7 [22]	72.2 [26]	
*Gestational Age (weeks)* ***	29.1 ± 1.8 [35]	40.2 ± 1.1 [39]	W^e^ = 1365, *p* < 0.001
*Sex* *			*p* = 0.022
Male	65.7 [23]	38.5 [15]	
Female	34.3 [12]	61.5 [24]	
*Birth Weight (g)* ***	1204 ± 339 [35]	3665 ± 513 [39]	*t* = -24.547, (CI: -2.661 to -2.260), df = 66.31, *p* < 0.001

Statistically significant differences were assessed by using t-test, Fisher’s exact test, χ^2^ test, or the Mann-Whitney U test. Statistics are only shown for significant associations.*p* < 0.1

*p < 0.05

**p < 0.01

***p< 0.001

^a^ n may differ for particular variables due to missing data

^b^ fetus exposed to maternal smoking

^c^ at least some tertiary education

^d^ DEPCAT greater than 3

^e^ W is the test statistic for the Wilcoxon Signed Rank Test, defined as the smaller of the sum of the positive ranks and the sum of the negative ranks.

### Telomere length comparisons: Preterm and term groups

**Fig 1:**
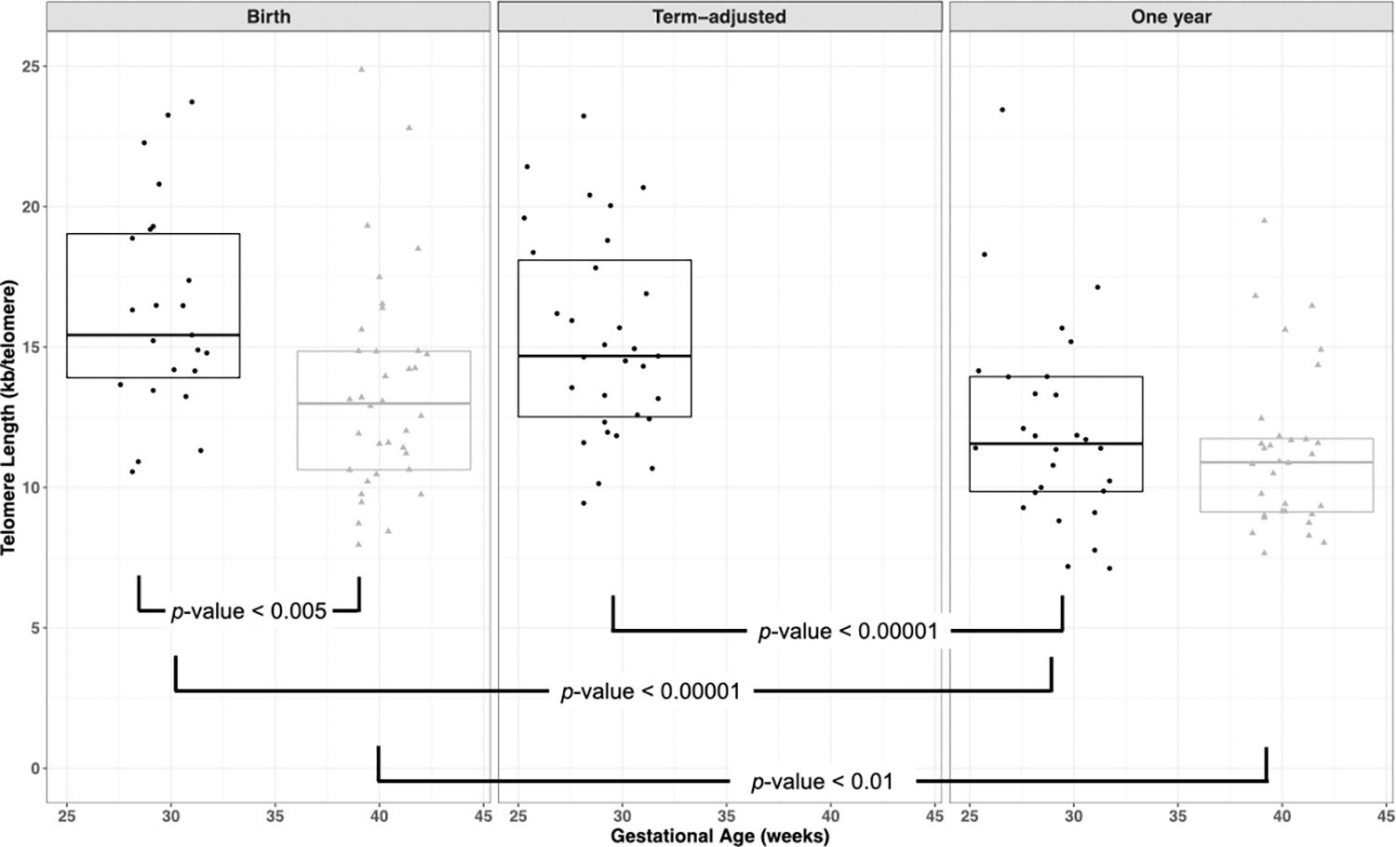
Telomere length is negatively associated with gestational age at birth, but not by one year of age. Absolute telomere length vs gestational age at birth (left panel), term-adjusted age (middle panel) and one year of age (right panel). Black circles and gray triangles represent samples from preterm and term individuals, respectively. Horizontal black lines represent the median TL for each group and boxes represent the interquartile range. Significant differences in TL as determined by *t* test are indicated.

Preterm infants had significantly longer telomeres than term infants at birth (mean difference of 2.9 kb/telomere, *p* < 0.005, n = 36 and 23, respectively for term and preterm infants; Fig 1; Table 2). Moreover, the mean telomere length of preterm infants remained longer at term-adjusted age than the term infant telomere lengths at birth (mean difference of 1.9 kb/telomere, t = 2.347, CI: 0.021 to 0.264, df = 64.97, *p* = 0.022, n = 36 and 31, respectively for term and preterm infants). The difference between telomere length at birth and term-adjusted age for the preterm infants was not significant (paired t-test: *t* = 1.240, df = 19, *p* = 0.230, n = 20; power analysis: power = 0.8, effect size = 0.58, alpha = 0.05). By one year corrected full term age, however, telomere lengths between the term and preterm groups were no longer significantly different (*t* = 0.9657, df = 53.08, *p* = 0.339, n = 32 and 28, respectively for term and preterm infants; Table 2).

**Table 2:** Telomere length and telomere shortening summary statistics for infants by preterm and term birth.

	Mean ± SD [n] ^*a*^		
Variable	Preterm	Term	Statistics
***Telomere Length (kb)*** ^***b***^			
Birth**	16.34 ± 3.76 [23]	13.44 ± 3.80 [36]	*t* = 3.164 (CI: 0.075 to 0.336), df = 51.93, *p* = 0.003
Term-adjusted age	15.36 ± 3.59 [31]	NA	
One year	12.14 ± 3.58 [28]	11.27 ± 2.85 [32]	
***Telomere Shortening (kb)***			
Birth to one year*	5.17 ± 3.75 [18]	2.56 ± 2.41 [30]	*t* = 2.644 (CI: 0.580 to 4.642), df = 25.56, *p* = 0.014
Birth to term-adjusted age	1.16 ± 4.30 [20]	NA	
Term-adjusted age to one year	3.66 ± 3.05 [25]	NA	

Statistically significant differences were assessed by using *t* tests.

*p < 0.05

**p < 0.01

^a^ n may differ for individual variables due to missing data

^b^ Telomere length was natural log-transformed prior to analysis. Telomere shortening was calculated using the untransformed telomere length data.

**Fig 2:**
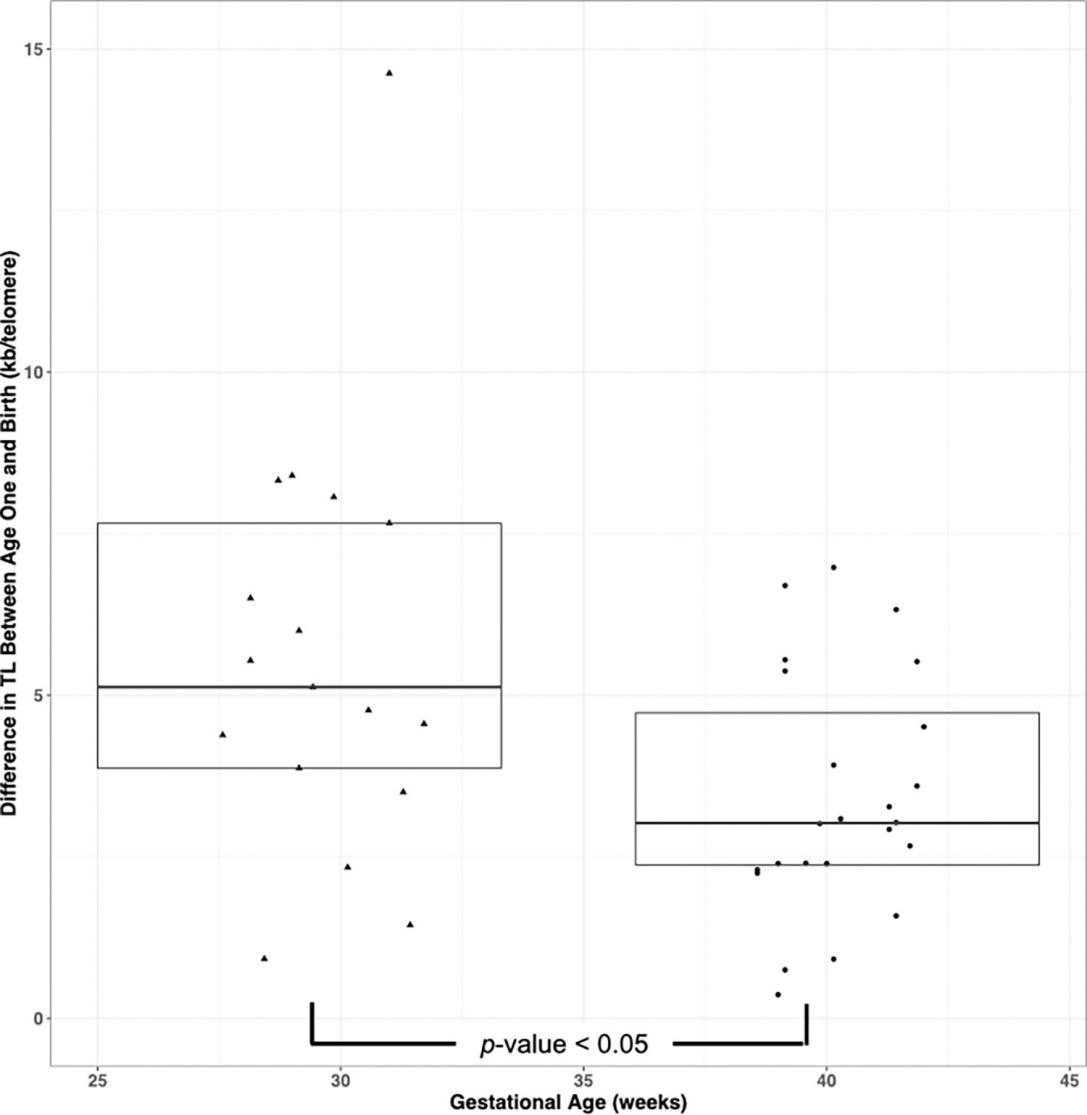
Telomere shortening is greater in preterm infants than term infants. The difference in telomere length between age one and birth vs gestational age at birth in preterm (triangles) and term (circles) infants. Horizontal black lines represent the median TL difference and boxes represent the interquartile range for each group. Significant difference in TL shortening as determined by *t* test is indicated.

Telomeres were significantly shorter in both the term and the preterm groups at one year of age compared with birth (Table 2, average difference of 4.3 kb/diploid genome, *t* = 6.223, df = 17, *p* < .001 and 2.0 kb/telomere, *t* = 5.642, df = 29, *p* < 0.001, respectively for the preterm (n = 18) and term (n = 30) infants) and post-natal telomere shortening was more rapid in the preterm group (5.2 vs 2.6 kb/telomere/year, *t* = 2.644, df = 25.56, *p* = 0.014; Fig 2, Tables 2 and 3) during the first year of life. While TL at birth was also inversely correlated with birth weight (*r* = 0.306, df = 57, *p* = 0.018), this is not the case when using the birthweight *z*-score (r = -0.006, df = 57, *p* = 0.962), suggesting that the correlation between birth weight and TL is due to the very high correlation between GA and birthweight. The correlation between the telomere length at birth and one year for all infants was 0.55 (*p* < 0.001, df = 46). This was likely driven by the term infants because the correlation between telomere length at birth and one year of age for term infants was 0.634 (*p* < .001, df = 28) but was lower (0.374, df = 16) and not statistically significant (*p* = 0.126) in preterm infants.

**Table 3:** Paired *t* test comparison of TL.

	Preterm			Term
	Birth to one year**	Birth to term-adjusted age	Term-adjusted age to one year**	Birth to one year**
Mean of the differences	0.361	0.072	0.274	0.205
*t*-statistic	6.223	1.240	6.335	5.642
95% Confidence Interval	0.239 to 0.483	0.049 to 0.192	0.185 to 0.363	0.130 to 0.279
Degrees of freedom	17	19	24	29

TLs were natural log transformed prior to analysis.

**p < 0.00001

### Effect of maternal health and sociodemographic factors on TL

#### TL at birth and corrected full-term age

Preterm infants had longer telomeres at birth than term infants. After adjusting for gestational age across the entire cohort, maternal chronic illness was negatively associated with telomere length at birth (β = -0.159, SE = 0.071, *p* = 0.029 n = 56). Maternal chronic illness was associated with shorter telomeres at birth if analysis was limited to preterm infants (β = -0.215, SE = 0.088, *p* = 0.024 n = 23). and remained associated after adjusting for gestational age (β = -0.215, SE = 0.091, *p* = 0.027 n = 23). This was not observed if analysis was limited to term infants (β = -0.106, SE = 0.272, *p* = 0.316, n = 33). The age of the mothers of preterm infants at birth was positively associated with telomere length at birth (β = 0.020, SE = 0.007, *p* = 0.006 n = 23) and term-adjusted age (β = 0.016, SE = 0.007, *p* = 0.021 n = 31). These results were robust to adjustment for gestational age (maternal age and TL at birth: β = 0.020, SE = 0.007, *p* = 0.008, n = 23; maternal age at birth and TL at term-adjusted age: β = 0.015, SE = 0.006, *p* = 0.019, n = 31). Maternal age also remained significantly associated with telomere length at birth and corrected term-adjusted age after including maternal chronic illness (β = 0.017, SE = 0.007, *p* = 0.020, n = 23; corrected term-adjusted age: (β = 0.016, SE = 0.006, *p* = 0.015 n = 30) or maternal education in the models (β = 0.023, SE = 0.007, *p* = 0.004, n = 23; corrected term-adjusted age: (β = 0.013, SE = 0.006, *p* = 0.047 n = 31). The full models for TL at birth or term-adjusted age in preterm infants are shown in Table 4 and all incremental models for TL at birth or term-adjusted infants are shown in S1 and S2 Tables, respectively. There was no significant association between any of the variables listed in Table 1 and TL at birth when analysis was limited to the term infants. There was no significant association with socioeconomic status. Similar results were observed if *z*-scored birth weight was used in lieu of gestational age (S3 and S4 Tables). The notable exceptions were associations between maternal chronic illness and TL. While not significantly associated with TL at birth in the entire cohort (β = -0.136, SE = 0.076, *p* = 0.080, adjusted *R*^2^ = 0.038, model *p* = 0.079, n = 56), maternal chronic illness was significantly associated with shorter telomeres at birth when analysis was limited to preterm infants (*β* = -0.215, SE = 0.088, *p* = 0.024, adjusted *R*^2^ = 0.183, *p* value of the model = 0.024, n = 56). Maternal chronic illness remained marginally associated with shorter telomeres at birth after adjusting for gestational age (β = -0.204, SE = 0.096, *p* = 0.046, adjusted *R*^2^ = 0.148, model *p* = 0.083) or *z*-scored birth weight (β = -0.204, SE = 0.096 *p* = 0.046, adjusted *R*^2^ = 0.148, *p* value of the model = 0.077, n = 23).

**Table 4:** Regression results for telomere length at birth in preterm infants.

	Birth	Term-adjusted Age
	Model A**	Model B**
**Constant**	1.595	3.710***
	(1.369)	(0.735)
	[0.188]	[< 0.001]
**Chronic Illness (Mother)**	-0.144	-0.007
	(0.086)	(0.078)
	[0.111]	[0.926]
**Gestational Age**	0.021	-0.048**
	(0.037)	(0.024)
	[0.571]	[0.039]
**Maternal Age**	0.019**	0.015**
	(0.007)	(0.007)
	[0.016]	[0.037]
**Post-secondary Education (Mother)**	-0.110	0.100
	(0.128)	(0.106)
	[0.401]	[0.356]
**R-squared**	0.442	0.325
**Adjusted R-squared**	0.318	0.216
**Model *p* value**	0.026	0.038
**No. observations**	23	30

Standard errors are reported in parentheses, *p*-values are in brackets.

*, **, *** indicate significance at the 90%, 95% and 99% level, respectively

#### Telomere length at year one

Statistically significant associations between the variables examined and TL at age one were only observed for the preterm group. Gestational age was negatively associated with telomere length (β = -0.07, SE = 0.025, *p* = 0.015, adjusted *R*^2^ = 0.177, *p* value of the model = 0.0149, n = 28). Lower birthweight was associated with longer telomeres (β = -0.352, SE = 0.147, *p* = 0.024, adjusted R^2^ = 0.149, model *p* value = 0.0242, n = 28), but this was not observed when *z*-scored birthweight was used (to adjust for gestational length; β = -0.051, SE = 0.057, *p* = 0.385, adjusted *R*^2^ = -0.008, *p* value of the model = 0.385, n = 28).

## Discussion

Telomere length was measured in genomic DNA from buccal swabs collected from very preterm infants during the first week after birth, at term-adjusted age and at year one. These values were compared with telomere lengths of genomic DNA isolated from buccal swabs collected from apparently healthy term infants during the first week after birth and at one year.

As described previously [48], risk factors for premature birth, high booking BMI, male fetal sex, fetal growth restriction [49], and exposure to cigarette smoke [50, 51], were enriched in the preterm group of infants compared with the term group (Table 1). These risk factors are also associated with shorter telomere lengths [52–55]. Compared with mothers of term infants, mothers of preterm infants were less likely to have post-secondary education or live in an affluent neighborhood. The mothers of term children were older than those of preterm children in contrast to what has been previously described [48], however this was attributed to recruitment.

Consistent with previous cross-sectional studies in leukocytes from venous [47, 56] or cord blood [57, 58], we found that saliva-derived telomere lengths were longer in preterm infants (both at birth and at term-adjusted age) compared with term infants at birth. Telomere shortening was more rapid in the preterm cohort than in term infants. In contrast with a previous study [47], the shortening rate in the preterm group was not significantly associated with gestational age or birthweight. This may be due to the larger sample size of the current study (n = 16 compared with n = 5), technical differences [59], or that this study utilized DNA from buccal cells whereas the previous study used blood. While data from a cross-sectional study suggests that the rate of telomere shortening is similar in neutrophils and T cells [60], it is possible that telomere shortening rates differ in the various cell populations (depending on cell replication rates and telomerase activity) comprising buccal samples and this may affect the ability to detect such a difference.

A few studies have compared differences in telomere length in groups of children or adolescents born prematurely or at term [61, 62]. Neither Hadchouel *et al*. (2015) nor Henckel *et al*. (2018) found statistically significant differences in telomere length measured from samples taken approximately at age 10 or 14.9 years, respectively. Indeed, in our study, the difference in telomere length between the samples collected at one year of age did not differ between the term or preterm groups in this study. The sample size was sufficient to detect longer telomeres in the preterm group at birth (term), term-adjusted (preterm) or one year at a medium effect size d (0.65) with a 5% Type I error and power of 0.80. While this is not the first study to examine telomere length in buccal samples from preterm infants [63], this is the first to demonstrate that telomere lengths in buccal samples collected from term infants at birth were shorter than telomere lengths from preterm infants collected at birth and term-adjusted age.

Evidence suggests that the intrauterine environment influences newborn TL and our study confirms that maternal health is associated with TL among preterm but not (in our study) in term births. TL in cord blood from infants born to mothers experiencing high psychosocial stress during pregnancy were shorter than infants born to low-stress mothers [64–66]. Another study, consisting of 1026 mother-infant pairs, demonstrated a negative association between socioeconomic status (SES) and cord blood TL [67]. Maternal folate level is positively associated [68], and maternal smoking has been negatively associated [69] with umbilical cord blood TL. In the current study, maternal smoking was not associated with shorter telomeres at birth, nor was it associated with more rapid TL shortening. There was also no difference between male and female newborns or DEPCAT status. Post-hoc power analysis indicated that this was likely due to sample size (S5 Table). All mothers in the study, except for three of the mothers of preterm infants, took folic acid during pregnancy and so it is difficult to assess a relationship between folic acid and TL. In contrast, preterm infants born to mothers experiencing chronic illness had shorter telomeres. This is difficult to interpret, given that several conditions were defined as chronic illness in the study, including mental illness, type 2 diabetes, epilepsy, hypothyroidism, among others. Maternal age was positively associated with TL in preterm infants (Table 4). This is consistent with the findings of Vasu *et al*. [47] and Okuda *et al*. [70]. This finding was robust to the inclusion of maternal education and maternal chronic illness in the model, indicating that the relationship between TL at birth and maternal age cannot be explained by socioeconomic factors.

Additional research examining the relationship between environment and TL in neonates, especially those born prematurely, is needed. Although still relatively small, our study consists of one of the largest cohorts with TL measured longitudinally at birth and one year for term and preterm infants and term-adjusted age for preterm infants. Our findings indicate more rapid TL shortening in preterm infants, perhaps reflecting that birth occurred prior to a late term burst of growth and cellular replication. Future research should aim to identify the biological processes behind these findings.

## Materials and methods

### Study participants

A cohort of 50 preterm infants (< 32 weeks gestation) and 40 term control infants (37–42 weeks gestation) were recruited during the first week of age from the Simpson Centre for Reproductive Health, Edinburgh, UK Royal Infirmary of Edinburgh as previously described [48]. Most of the parents of the term babies were approached prior to delivery. None of the term babies had suspected or proven fetal anomaly or proven infection. The term infants were apparently healthy with the exception of one who had jaundice. The term babies stayed in the hospital for an average of 4.5 days (range 2–9) with their mothers. Ethical approval was obtained from the South East Scotland Research Ethics Committee (Reference 11/AL/0329). NHS management approval was obtained (Lothian R&D Project number 2011/R/ NE/03). Infant samples were collected under the framework of the Edinburgh Reproductive Tissue BioBank (West of Scotland Research Ethics Service Reference 09/S0704/3) following an amendment to ethical approval (Reference AM07/1). All parents gave written informed consent, and all studies were performed in accordance with the Declaration of Helsinki. Term controls were born at least 37 completed weeks post last menstrual period (LMP) with no identified maternal or fetal complications. In the control group, only women with singleton pregnancies, without pre-existing hypertension or diabetes and who were non-smokers in the current pregnancy were included. Demographic and clinical data were collected from hospital and research visits and hospital records. From the main cohort, there were 32 preterm and 39 term infants with available DNA for the TL assay. The characteristics of this smaller group are described in Table 1. Socioeconomic status is approximated using deprivation category (DEPCAT) scores derived from the Carstairs score of the subject’s postal code [71]. The DEPCAT scores are categorical variables ranging from 1–7 with 1 and 2 being the most affluent.

### Sample collection

Saliva for DNA was collected from the preterm infants at birth; at term-adjusted age); and at one year corrected; and from term infants at birth and one year of age. Samples were collected from preterm infants within a median of 3 days (interquartile range (IQR) of 1.75–4 days from birth) and term infants within a median of 2 days (IQR of 1–2.3 days) from birth. Saliva was collected using the Oragene DNA (OG-250) kits and saliva sponges CS-1 and extracted using prepIT-2LP (DNA Genotek, Ottawa, ON, Canada). DNA was quantified using the Qubit 2.0 Fluorometer (Life Technologies, Paisley, UK) and stored at -20°C until received by the Notterman laboratory, where it was stored at -80°C.

### Telomere length

TL was measured by absolute quantitative real-time PCR (qPCR) [72–75]. Two double stranded oligonucleotides (Integrated DNA Technologies), an 84-mer consisting of (TTAGG)_16_ and a 79-mer containing sequence from the *36B4* gene were used to construct standard curves to determine absolute telomere length and number of diploid genomes copies, respectively. TL and single copy gene qPCR assays were performed on separate plates. Each sample was assayed in triplicate and the results averaged. Individual TL was determined by dividing the telomere length per genome by 92, the number of telomeres per diploid genome. Each plate contained DNA from a cell line with a relatively short telomere length (3C167b) [76] and a fibroblast cell line containing a stable integration of TERT, which encodes the protein component of telomerase (NHFpreT) [77]. These were used to control for inter-plate variation as described [73, 74]. Human genomic DNA was also included to determine the coefficient of variation (0.09) [73, 74]. The intraclass correlation coefficients (calculated using the Ct values) were 0.975 (CI 0.968–0.981) and 0.949 (CI 0.934–0.961), respectively for the telomere and 36B4 technical replicates.

### Statistical analysis

Power analysis was performed with G*Power version 3.1.9.6 [78, 79] and the R pwr package [80]. All other statistical analysis was performed using R version 4.0.5 [81]. Body mass index was analyzed as either a continuous variable or converted to a categorical variable. Data were tested for normality using the Shapiro-Wilk test. The primary outcome, telomere length, was not normally distributed and was natural log transformed for analysis. One percent was trimmed off both tails of the sample to remove outliers. After transformation, data was normally distributed. Telomere shortening was calculated by subtracting telomere length at term age (or age one from telomere length at birth or term age as indicated. Differences were calculated using untransformed telomere length value. Telomere shortening values were normally distributed.

## Supporting information

S1 Table Regression results for telomere length at birth in preterm infants. (DOCX)

S2 Table Regression results for telomere length at term-adjusted age in preterm infants. (DOCX)

S3 Table Regression results for telomere length at birth in preterm infants. (DOCX)

S4 Table Regression results for telomere length at corrected full-term age in preterm infants. (DOCX)

S5 Table Post-hoc power analysis. (DOCX)

## References

[1] World Health Organization. WHO: recommended definitions, terminology and format for statistical tables related to the perinatal period and use of a new certificate for cause of perinatal deaths. Modifications recommended by FIGO as amended October 14, 1976. Acta Obstet Gynecol Scand 1977;56:247–53. 560099

[2] Information Services Division (ISD). Scotland. Births in Scottish hospitals: Year ending March 2019. Scotland: 2019.

[3] Blencowe H, Cousens S, Oestergaard MZ, Chou D, Moller AB, Narwal R, et al. National, regional, and worldwide estimates of preterm birth rates in the year 2010 with time trends since 1990 for selected countries: a systematic analysis and implications. Lancet 2012;379:2162–72. doi: 10.1016/S0140-6736(12)60820-4 22682464

[4] Zeitlin J, Szamotulska K, Drewniak N, Mohangoo AD, Chalmers J, Sakkeus L, et al. Preterm birth time trends in Europe: a study of 19 countries. BJOG 2013;120:1356–65. doi: 10.1111/1471-0528.12281 23700966 PMC4285908

[5] Chang HH, Larson J, Blencowe H, Spong CY, Howson CP, Cairns-Smith S, et al. Preventing preterm births: analysis of trends and potential reductions with interventions in 39 countries with very high human development index. Lancet 2013;381:223–34. doi: 10.1016/S0140-6736(12)61856-X 23158883 PMC3572865

[6] Shay JW, Wright WE. Telomeres and telomerase: three decades of progress. Nat Rev Genet 2019;20:299–309. doi: 10.1038/s41576-019-0099-1 30760854

[7] Bodnar AG, Ouellette M, Frolkis M, Holt SE, Chiu CP, Morin GB, et al. Extension of life-span by introduction of telomerase into normal human cells. Science 1998;279:349–52. doi: 10.1126/science.279.5349.349 9454332

[8] Hayflick L, Moorhead PS. The serial cultivation of human diploid cell strains. Exp Cell Res 1961;25:585–621. doi: 10.1016/0014-4827(61)90192-6 13905658

[9] Allsopp RC, Vaziri H, Patterson C, Goldstein S, Younglai EV, Futcher AB, et al. Telomere length predicts replicative capacity of human fibroblasts. Proc Natl Acad Sci U S A 1992;89:10114–8. doi: 10.1073/pnas.89.21.10114 1438199 PMC50288

[10] Turner KJ, Vasu V, Griffin DK. Telomere biology and human phenotype. Cells 2019;8:E73. doi: 10.3390/cells8010073 30669451 PMC6356320

[11] Marioni RE, Harris SE, Shah S, McRae AF, von Zglinicki T, Martin-Ruiz C, et al. The epigenetic clock and telomere length are independently associated with chronological age and mortality. Int J Epidemiol 2018;47:356. doi: 10.1093/ije/dyx233 29190382 PMC5837660

[12] Harley CB, Futcher AB, Greider CW. Telomeres shorten during ageing of human fibroblasts. Nature 1990;345:458–60. doi: 10.1038/345458a0 2342578

[13] Notterman DA, Schneper L. Telomere time—Why we should treat biological age cautiously. JAMA Netw Open 2020;3:e204352. doi: 10.1001/jamanetworkopen.2020.4352 32364591

[14] Artandi SE, DePinho RA. Telomeres and telomerase in cancer. Carcinogenesis 2010;31:9–18. doi: 10.1093/carcin/bgp268 19887512 PMC3003493

[15] Atturu G, Brouilette S, Samani NJ, London NJM, Sayers RD, Bown MJ. Short leukocyte telomere length is associated with abdominal aortic aneurysm (AAA). Eur J Vasc Endovasc Surg 2010;39:559–64. doi: 10.1016/j.ejvs.2010.01.013 20172749

[16] Aubert G, Lansdorp PM. Telomeres and aging. Physiol Rev 2008;88:557–79. doi: 10.1152/physrev.00026.2007 18391173

[17] Epel ES, Blackburn EH, Lin J, Dhabhar FS, Adler NE, Morrow JD, et al. Accelerated telomere shortening in response to life stress. Proc Natl Acad Sci U S A 2004;101:17312–5. doi: 10.1073/pnas.0407162101 15574496 PMC534658

[18] Epel ES, Merkin SS, Cawthon R, Blackburn EH, Adler NE, Pletcher MJ, et al. The rate of leukocyte telomere shortening predicts mortality from cardiovascular disease in elderly men. Aging (Albany NY) 2008;1:81–8. doi: 10.18632/aging.100007 20195384 PMC2830080

[19] Farzaneh-Far R, Cawthon RM, Na B, Browner WS, Schiller NB, Whooley MA. Prognostic value of leukocyte telomere length in patients with stable coronary artery disease: data from the Heart and Soul Study. Arterioscler Thromb Vasc Biol 2008;28:1379–84. doi: 10.1161/ATVBAHA.108.167049 18467646 PMC2675880

[20] Fitzpatrick AL, Kronmal RA, Kimura M, Gardner JP, Psaty BM, Jenny NS, et al. Leukocyte telomere length and mortality in the Cardiovascular Health Study. J Gerontol A Biol Sci Med Sci 2011;66:421–9. doi: 10.1093/gerona/glq224 21289018 PMC3055278

[21] Kodali HP, Borrell LN. Telomere length and mortality risk among adults in the United States: The role of age and race/ethnicity. Ann Epidemiol 2021:S1047–2797(21)00229-5. 10.1016/j.annepidem.2021.07.013. 34343614

[22] Miner AE, Graves JS. What telomeres teach us about MS. Mult Scler Relat Disord 2021;54:103084. doi: 10.1016/j.msard.2021.103084 34371369

[23] Njajou OT, Hsueh W-C, Blackburn EH, Newman AB, Wu S-H, Li R, et al. Association between telomere length, specific causes of death, and years of healthy life in health, aging, and body composition, a population-based cohort study. J Gerontol A Biol Sci Med Sci 2009;64:860–4. doi: 10.1093/gerona/glp061 19435951 PMC2981462

[24] Oeseburg H, de Boer RA, van Gilst WH, van der Harst P. Telomere biology in healthy aging and disease. Pflugers Arch 2010;459:259–68. doi: 10.1007/s00424-009-0728-1 19756717 PMC2801851

[25] Risques RA, Arbeev KG, Yashin AI, Ukraintseva SV, Martin GM, Rabinovitch PS, et al. Leukocyte telomere length is associated with disability in older U.S. population. J Am Geriatr Soc 2010;58:1289–98. doi: 10.1111/j.1532-5415.2010.02948.x 20579170 PMC2918372

[26] Vasan RS, Demissie S, Kimura M, Cupples LA, Rifai N, White C, et al. Association of leukocyte telomere length with circulating biomarkers of the renin-angiotensin-aldosterone system: The Framingham Heart Study. Circulation 2008;117:1138–44. doi: 10.1161/CIRCULATIONAHA.107.731794 18268147 PMC3142671

[27] Willeit P, Raschenberger J, Heydon EE, Tsimikas S, Haun M, Mayr A, et al. Leucocyte telomere length and risk of type 2 diabetes mellitus: New prospective cohort study and literature-based meta-analysis. PLoS One 2014;9:e112483. doi: 10.1371/journal.pone.0112483 25390655 PMC4229188

[28] Zheng X, Wezel F, Azoitei A, Meessen S, Wang W, Najjar G, et al. Shorter leukocyte telomere length Is associated with worse survival of patients with bladder cancer and renal cell carcinoma. Cancers (Basel) 2021;13:3774. doi: 10.3390/cancers13153774 34359672 PMC8345040

[29] Azcona-Sanjulian MC. Telomere length and pediatric obesity: A review. Genes (Basel) 2021;12:946. doi: 10.3390/genes12060946 34205609 PMC8233934

[30] Garfein J, Flannagan KS, Rittman D, Ramirez-Zea M, Villamor E, Nine Mesoamerican Countries Metabolic Syndrome Study (NiMeCoMeS) Group. Leukocyte telomere length is inversely associated with a metabolic risk score in Mesoamerican children. Am J Hum Biol 2021:e23596. 10.1002/ajhb.23596. 33720476

[31] Gianesin K, Noguera-Julian A, Zanchetta M, Del Bianco P, Petrara MR, Freguja R, et al. Premature aging and immune senescence in HIV-infected children. AIDS 2016;30:1363–73. doi: 10.1097/QAD.0000000000001093 26990630 PMC4867984

[32] Guttmacher AE, Raju TNK. The child is father of the man, and mother of the woman. Pediatrics 2014;134:e1411–1412. doi: 10.1542/peds.2014-2646 25349322 PMC8194474

[33] Kaali S, Jack D, Opoku-Mensah J, Bloomquist T, Aanaro J, Quinn A, et al. Prenatal household air pollution exposure, cord blood mononuclear cell telomere length and age four blood pressure: Evidence from a Ghanaian pregnancy cohort. Toxics 2021;9:169. doi: 10.3390/toxics9070169 34357912 PMC8309911

[34] Shah A, George M, Dhangar S, Rajendran A, Mohan S, Vundinti BR. Severe telomere shortening in Fanconi anemia complementation group L. Mol Biol Rep 2021;48:585–93. doi: 10.1007/s11033-020-06101-2 33394227

[35] Shalev I, Entringer S, Wadhwa PD, Wolkowitz OM, Puterman E, Lin J, et al. Stress and telomere biology: a lifespan perspective. Psychoneuroendocrinology 2013;38:1835–42. doi: 10.1016/j.psyneuen.2013.03.010 23639252 PMC3735679

[36] Suliman ME, Ansari MGA, Rayis MA, Hamza MA, Saeed AA, Mohammed AK, et al. Telomere length and telomere repeat-binding protein in children with sickle cell disease. Pediatr Res 2021. doi: 10.1038/s41390-021-01495-6 33824452 PMC8904250

[37] Tatsi C, Flippo C, Faucz FR, Sinaii N, Stratakis CA. Telomere length changes in children with Cushing disease: A pilot study. J Endocr Soc 2020;4:bvaa067. doi: 10.1210/jendso/bvaa067 32666011 PMC7343236

[38] Tung KTS, Wong RS, Tsang H-W, Chua GT, Chan D, Chan KC, et al. Impact of snoring on telomere shortening in adolescents with atopic diseases. Genes (Basel) 2021;12:766. doi: 10.3390/genes12050766 34069972 PMC8157836

[39] Hanssen LM, Schutte NS, Malouff JM, Epel ES. The relationship between childhood psychosocial stressor level and telomere length: A meta-analysis. Health Psychol Res 2017;5:6378. doi: 10.4081/hpr.2017.6378 28603779 PMC5452631

[40] Lang J, McKie J, Smith H, McLaughlin A, Gillberg C, Shiels PG, et al. Adverse childhood experiences, epigenetics and telomere length variation in childhood and beyond: A systematic review of the literature. Eur Child Adolesc Psychiatry 2020;29:1329–38. doi: 10.1007/s00787-019-01329-1 30968208 PMC7501093

[41] Pepper GV, Bateson M, Nettle D. Telomeres as integrative markers of exposure to stress and adversity: A systematic review and meta-analysis. R Soc Open Sci 2018;5:180744. doi: 10.1098/rsos.180744 30225068 PMC6124068

[42] Schutte NS, Malouff JM. The relationship between perceived stress and telomere length: A meta-analysis. Stress Health 2016;32:313–9. doi: 10.1002/smi.2607 25393133

[43] Shalev I. Early life stress and telomere length: Investigating the connection and possible mechanisms: a critical survey of the evidence base, research methodology and basic biology. Bioessays 2012;34:943–52. doi: 10.1002/bies.201200084 22991129 PMC3557830

[44] Smith L, Luchini C, Demurtas J, Soysal P, Stubbs B, Hamer M, et al. Telomere length and health outcomes: An umbrella review of systematic reviews and meta-analyses of observational studies. Ageing Res Rev 2019;51:1–10. doi: 10.1016/j.arr.2019.02.003 30776454

[45] Vaiserman A, Krasnienkov D. Telomere length as a marker of biological age: State-of-the-art, open issues, and future perspectives. Front Genet 2020;11:630186. doi: 10.3389/fgene.2020.630186 33552142 PMC7859450

[46] Friedrich U, Schwab M, Griese EU, Fritz P, Klotz U. Telomeres in neonates: New insights in fetal hematopoiesis. Pediatr Res 2001;49:252–6. doi: 10.1203/00006450-200102000-00020 11158522

[47] Vasu V, Turner KJ, George S, Greenall J, Slijepcevic P, Griffin DK. Preterm infants have significantly longer telomeres than their term born counterparts. PLoS One 2017;12:e0180082. doi: 10.1371/journal.pone.0180082 28658264 PMC5489189

[48] Piyasena C, Cartier J, Provençal N, Wiechmann T, Khulan B, Sunderesan R, et al. Dynamic changes in DNA methylation occur during the first year of life in preterm infants. Front Endocrinol (Lausanne) 2016;7:158. doi: 10.3389/fendo.2016.00158 28018293 PMC5156662

[49] Gardosi JO. Prematurity and fetal growth restriction. Early Hum Dev 2005;81:43–9. doi: 10.1016/j.earlhumdev.2004.10.015 15707714

[50] Shah NR, Bracken MB. A systematic review and meta-analysis of prospective studies on the association between maternal cigarette smoking and preterm delivery. Am J Obstet Gynecol 2000;182:465–72. doi: 10.1016/s0002-9378(00)70240-7 10694353 PMC2706697

[51] Simpson WJ. A preliminary report on cigarette smoking and the incidence of prematurity. Am J Obstet Gynecol 1957;73:807–15. 13411046

[52] Martens DS, Plusquin M, Gyselaers W, De Vivo I, Nawrot TS. Maternal pre-pregnancy body mass index and newborn telomere length. BMC Med 2016;14:148. doi: 10.1186/s12916-016-0689-0 27751173 PMC5067896

[53] Aubert G, Baerlocher GM, Vulto I, Poon SS, Lansdorp PM. Collapse of telomere homeostasis in hematopoietic cells caused by heterozygous mutations in telomerase genes. PLoS Genet 2012;8:e1002696. doi: 10.1371/journal.pgen.1002696 22661914 PMC3355073

[54] Davy P, Nagata M, Bullard P, Fogelson NS, Allsopp R. Fetal growth restriction is associated with accelerated telomere shortening and increased expression of cell senescence markers in the placenta. Placenta 2009;30:539–42. doi: 10.1016/j.placenta.2009.03.005 19359039 PMC2692289

[55] Wei H, Zhang C, Silveyra P. The relationships between prenatal smoking exposure and telomere lengths in fetuses, infants, and children: A systematic literature review. J Addict Nurs 2020;31:243–52. doi: 10.1097/JAN.0000000000000364 33264196

[56] Holmes DK, Bellantuono I, Walkinshaw SA, Alfirevic Z, Johnston TA, Subhedar NV, et al. Telomere length dynamics differ in foetal and early post-natal human leukocytes in a longitudinal study. Biogerontology 2009;10:279–84. doi: 10.1007/s10522-008-9194-y 18989747

[57] Menon R, Yu J, Basanta-Henry P, Brou L, Berga SL, Fortunato SJ, et al. Short fetal leukocyte telomere length and preterm prelabor rupture of the membranes. PLoS One 2012;7:e31136. doi: 10.1371/journal.pone.0031136 22348044 PMC3278428

[58] Sibert NT, Ventura Ferreira MS, Wagner W, Eipel M, Dreschers S, Brümmendorf TH, et al. Cord blood telomere shortening associates with increased gestational age and birth weight in preterm neonates. Exp Ther Med 2021;21:344. doi: 10.3892/etm.2021.9775 33732317 PMC7903469

[59] Turner KJ, Vasu V, Greenall J, Griffin DK. Telomere length analysis and preterm infant health: the importance of assay design in the search for novel biomarkers. Biomark Med 2014;8:485–98. doi: 10.2217/bmm.14.13 24796612

[60] Robertson JD, Gale RE, Wynn RF, Dougal M, Linch DC, Testa NG, et al. Dynamics of telomere shortening in neutrophils and T lymphocytes during ageing and the relationship to skewed X chromosome inactivation patterns. Br J Haematol 2000;109:272–9. doi: 10.1046/j.1365-2141.2000.01970.x 10848812

[61] Hadchouel A, Marchand-Martin L, Franco-Montoya ML, Peaudecerf L, Ancel PY, Delacourt C, et al. Salivary telomere length and lung function in adolescents born very preterm: A prospective multicenter study. PLoS One 2015;10:e0136123. doi: 10.1371/journal.pone.0136123 26355460 PMC4565668

[62] Henckel E, Svenson U, Nordlund B, Berggren Brostrom E, Hedlin G, Degerman S, et al. Telomere length was similar in school-age children with bronchopulmonary dysplasia and allergic asthma. Acta Paediatr 2018;107:1395–401. doi: 10.1111/apa.14294 29476624

[63] Casavant SG, Li H, Reese B, Chen M-H, Cong X. Pilot study of absolute telomere lengths in preterm infants. Nurs Res 2021. doi: 10.1097/NNR.0000000000000535 34173371 PMC8563375

[64] Entringer S, Epel ES, Lin J, Buss C, Shahbaba B, Blackburn EH, et al. Maternal psychosocial stress during pregnancy is associated with newborn leukocyte telomere length. Am J Obstet Gynecol 2013;208:134.e1–7. doi: 10.1016/j.ajog.2012.11.033 23200710 PMC3612534

[65] Marchetto NM, Glynn RA, Ferry ML, Ostojic M, Wolff SM, Yao R, et al. Prenatal stress and newborn telomere length. Am J Obstet Gynecol 2016;215:94.e1-8. doi: 10.1016/j.ajog.2016.01.177 26829506

[66] Send TS, Gilles M, Codd V, Wolf I, Bardtke S, Streit F, et al. Telomere length in newborns is related to maternal stress during pregnancy. Neuropsychopharmacology 2017;42:2407–13. doi: 10.1038/npp.2017.73 28397798 PMC5645750

[67] Martens DS, Van Der Stukken C, Derom C, Thiery E, Bijnens EM, Nawrot TS. Newborn telomere length predicts later life telomere length: Tracking telomere length from birth to child- and adulthood. EBioMedicine 2021;63:103164. doi: 10.1016/j.ebiom.2020.103164 33422989 PMC7808927

[68] Louis-Jacques AF, Salihu HM, King LM, Paothong A, Sinkey RG, Pradhan A, et al. A positive association between umbilical cord RBC folate and fetal TL at birth supports a potential for fetal reprogramming. Nutr Res 2016;36:703–9. doi: 10.1016/j.nutres.2016.01.009 27269132

[69] Liu B, Song L, Zhang L, Wu M, Wang L, Cao Z, et al. Prenatal second-hand smoke exposure and newborn telomere length. Pediatr Res 2020;87:1081–5. doi: 10.1038/s41390-019-0594-2 31578036

[70] Okuda K, Bardeguez A, Gardner JP, Rodriguez P, Ganesh V, Kimura M, et al. Telomere length in the newborn. Pediatr Res 2002;52:377–81. doi: 10.1203/00006450-200209000-00012 12193671

[71] McCloone P. Carstairs scores for Scottish postcode sectors from the 1991 census. University of Glasgow: 1994.

[72] Cawthon RM. Telomere measurement by quantitative PCR. Nucleic Acids Res 2002;30:e47. doi: 10.1093/nar/30.10.e47 12000852 PMC115301

[73] Mitchell C, Hobcraft J, McLanahan SS, Siegel SR, Berg A, Brooks-Gunn J, et al. Social disadvantage, genetic sensitivity, and children’s telomere length. Proc Natl Acad Sci U S A 2014;111:5944–9. doi: 10.1073/pnas.1404293111 24711381 PMC4000782

[74] Mitchell C, McLanahan S, Schneper L, Garfinkel I, Brooks-Gunn J, Notterman D. Father loss and child telomere length. Pediatrics 2017;140. doi: 10.1542/peds.2016-3245 28716823 PMC5527665

[75] O’Callaghan NJ, Fenech M. A quantitative PCR method for measuring absolute telomere length. Biol Proced Online 2011;13:3. doi: 10.1186/1480-9222-13-3 21369534 PMC3047434

[76] Wang S, Zhu J. Evidence for a relief of repression mechanism for activation of the human telomerase reverse transcriptase promoter. J Biol Chem 2003;278:18842–50. doi: 10.1074/jbc.M209544200 12611896

[77] Cheng, Zhao Y, Wang S, Zhang F, Russo M, McMahon SB, et al. Repression of telomerase gene promoter requires human-specific genomic context and is mediated by multiple HDAC1-containing corepressor complexes. FASEB J 2017;31:1165–78. doi: 10.1096/fj.201601111R 27940549 PMC5295725

[78] Faul F, Erdfelder E, Lang A-G, Buchner A. G*Power 3: a flexible statistical power analysis program for the social, behavioral, and biomedical sciences. Behav Res Methods 2007;39:175–91. doi: 10.3758/bf03193146 17695343

[79] Faul F, Erdfelder E, Buchner A, Lang A-G. Statistical power analyses using G*Power 3.1: tests for correlation and regression analyses. Behav Res Methods 2009;41:1149–60. doi: 10.3758/BRM.41.4.1149 19897823

[80] Champely S. pwr: Basic Functions for Power Analysis. 2020.

[81] R Core Team. R: A language and environment for statistical computing 2021.

