# Supplementary material for: Characteristics of salivary telomere length shortening in preterm infants
Source: PLoS One. 2023 Jan 17;18(1):e0280184. doi: 10.1371/journal.pone.0280184 (PMC9844854; doi:10.1371/journal.pone.0280184)
Supplement: S1 Table — (DOCX) [file pone.0280184.s001.docx]

**Supplemental Table 1: Regression results for telomere length at birth in preterm infants**

|  | **Model A**** | **Model B*** | **Model C***** | **Model D**** | **Model E**** | **Model F**** | **Model G**** |
| --- | --- | --- | --- | --- | --- | --- | --- |
| **Constant** | 2.853***  (0.055)  [< 0.001] | 2.772 ***  (1.075)  [0.018] | 2.130***  (0.214)  [< 0.001] | 1.973*  (1.049)  [0.074] | 1.246  (1.199)  [0.312] | 1.595  (1.369)  [0.188] | 2.117**  (0.987)  [0.045] |
| **Chronic Illness (Mother)** | -0.215**  (0.088)  [0.024] | -0.215**  (0.091)  [0.027] |  |  |  | -0.144  (0.086)  [0.111] | -0.160*  (0.08)  [0.070] |
| **Gestational Age** |  | 0.003  (0.036)  [0.941] |  | 0.005  (0.034)  [0.880] | 0.027  (0.038)  [0.488] | 0.021  (0.037)  [0.571] | 0.006  (0.032)  [0.852] |
| **Maternal Age** |  |  | 0.020***  (0.007)  [0.006] | 0.020***  (0.007)  [0.008] | 0.023***  (0.007)  [0.004] | 0.019**  (0.007)  [0.016] | 0.017**  (0.007)  [0.020] |
| **Post-secondary Education (Mother)** |  |  |  |  | -0.157  (0.130)  [0.241] | -0.110  (0.128)  [0.401] |  |
| **R-squared** | 0.220 | 0.221 | 0.305 | 0.305 | 0.355 | 0.442 | 0.419 |
| **Adjusted R-squared** | 0.183 | 0.143 | 0.272 | 0.236 | 0.253 | 0.318 | 0.327 |
| **Model *p* value** | 0.024 | 0.083 | 0.006 | 0.026 | 0.036 | 0.026 | 0.014 |
| **No. observations** | 23 | 23 | 23 | 23 | 23 | 23 | 23 |

Standard errors are reported in parentheses, *p*-values are in brackets. *, **, *** indicate significance at the 90%, 95% and 99% level, respectively
