# Supplementary material for: Characteristics of salivary telomere length shortening in preterm infants
Source: PLoS One. 2023 Jan 17;18(1):e0280184. doi: 10.1371/journal.pone.0280184 (PMC9844854; doi:10.1371/journal.pone.0280184)
Supplement: S2 Table — (DOCX) [file pone.0280184.s002.docx]

**Supplemental Table 2: Regression results for telomere length at term-adjusted age in preterm infants**

|  | **Model A** | **Model B** | **Model C**** | **Model D** | **Model E**** | **Model F**** | **Model G**** |
| --- | --- | --- | --- | --- | --- | --- | --- |
| **Constant** | 2.714***  (0.056)  [< 0.001] | 4.016 **  (0.689)  [< 0.001] | 2.206***  (0.209)  [< 0.001] | 3.45***  (0.648)  [< 0.001] | 3.784***  (0.710)  [< 0.001] | 3.710***  (0.735)  [< 0.001] | 3.420***  (0.666)  [< 0.001] |
| **Chronic Illness (Mother)** | -0.001  (0.088)  [0.989] | -0.025  (0.085)  [0.775] |  |  |  | -0.007  (0.078)  [0.926] | -0.006  (0.078)  [0.936] |
| **Gestational Age** |  | -0.044  (0.023)  [0.069]* |  | -0.042*  (0.021)  [0.054] | -0.052**  (0.023)  [0.029] | -0.048**  (0.024)  [0.039] | -0.042*  (0.021)  [0.060] |
| **Maternal Age** |  |  | 0.016**  (0.007)  [0.021] | 0.015**  (0.006)  [0.019] | 0.013**  (0.006)  [0.047] | 0.015**  (0.007)  [0.037] | 0.016**  (0.006)  [0.015] |
| **Post-secondary Education (Mother)** |  |  |  |  | 0.118  (0.104)  [0.267] | 0.100  (0.106)  [0.356] |  |
| **R-squared** | 0.000 | 0.1173 | 0.170 | 0.274 | 0.307 | 0.325 | 0.301 |
| **Adjusted R-squared** | -0.036 | 0.052 | 0.141 | 0.223 | 0.230 | 0.216 | 0.220 |
| **Model *p* value** | 0.989 | 0.186 | 0.021 | 0.011 | 0.018 | 0.038 | 0.024 |
| **No. observations** | 31 | 31 | 31 | 31 | 31 | 30 | 30 |

Standard errors are reported in parentheses, *p*-values are in brackets. *, **, *** indicate significance at the 90%, 95% and 99% level, respectively
