# Supplementary material for: Characteristics of salivary telomere length shortening in preterm infants
Source: PLoS One. 2023 Jan 17;18(1):e0280184. doi: 10.1371/journal.pone.0280184 (PMC9844854; doi:10.1371/journal.pone.0280184)
Supplement: S3 Table — (DOCX) [file pone.0280184.s003.docx]

**Supplementary Table 3: Regression results for telomere length at birth in preterm infants**

|  | **Model A**** | **Model B*** | **Model C***** | **Model D**** | **Model E**** | **Model F**** | **Model G**** |
| --- | --- | --- | --- | --- | --- | --- | --- |
| **Constant** | 2.853***  (0.055)  [< 0.001] | 2..856***  (0.057)  [< 0.001] | 2.130***  (0.214)  [< 0.001] | 2.155***  (0.213)  [< 0.001] | 2.108***  (0.221)  [< 0.001] | 2.250***  (0.236)  [< 0.001] | 2.292***  (0.222)  [< 0.001] |
| **Chronic Illness (Mother)** | -0.215**  (0.088)  [0.024] | -0.204**  (0.096)  [0.046] |  |  |  | -0.132  (0.090)  [0.161] | 0.142  (0.087)  [0.121] |
| **Z-scored Birth Weight** |  | 0.020  (0.054)  [0.714] |  | 0.056  (0.047)  [0.252] | 0.050  (0.048)  [0.300] | 0.028  (0.049)  [0.577] | 0.029  (0.048)  [0.548] |
| **Maternal Age** |  |  | 0.020***  (0.007)  [0.006] | 0.020***  (0.007)  [0.007] | 0.022***  (0.007)  [0.005] | 0.019**  (0.007)  [0.017] | 0.017**  (0.007)  [0.018] |
| **Post-secondary Education (Mother)** |  |  |  |  | -0.100  (0.114)  [0.391] | -0.071  (0.112)  [0.534] |  |
| **R-squared** | 0.220 | 0.226 | 0.305 | 0.350 | 0.375 | 0.442 | 0.429 |
| **Adjusted R-squared** | 0.183 | 0.148 | 0.272 | 0.285 | 0.277 | 0.318 | 0.339 |
| **Model *p* value** | 0.024 | 0.077 | 0.006 | 0.013 | 0.027 | 0.026 | 0.012 |
| **No. observations** | 23 | 23 | 23 | 23 | 23 | 23 | 23 |

Standard errors are reported in parentheses, *p*-values are in brackets. *, **, *** indicate significance at the 90%, 95% and 99% level, respectively.
