# Supplementary material for: Characteristics of salivary telomere length shortening in preterm infants
Source: PLoS One. 2023 Jan 17;18(1):e0280184. doi: 10.1371/journal.pone.0280184 (PMC9844854; doi:10.1371/journal.pone.0280184)
Supplement: S4 Table — (DOCX) [file pone.0280184.s004.docx]

**Supplementary Table 4: Regression results for telomere length at corrected full-term age in preterm infants**

|  | **Model A** | **Model B** | **Model C**** | **Model D**** | **Model E*** | **Model F** | **Model G*** |
| --- | --- | --- | --- | --- | --- | --- | --- |
| **Constant** | 2.714***  (0.056)  [< 0.001] | 2.726  (0.056)  [< 0.001] | 2.221***  (0.209)  [< 0.001] | 2.272***  (0.216)  [< 0.001] | 2.285***  (0.223)  [< 0.001] | 2.235***  (0.230)  [< 0.001] | 2.227***  (0.222)  [< 0.001] |
| **Chronic Illness (Mother)** | -0.001  (0.088)  [0.989] | 0.028  (0.089)  [0.758] |  |  |  | 0.036  (0.085)  [0.676] | 0.035  (0.083)  [0.680] |
| **Z-scored Birth Weight** |  | 0.059  (0.042)  [0.172] |  | 0.042  (0.038)  [0.280] | 0.043  (0.039)  [0.277] | 0.041  (0.041)  [0.326] | 0.040  (0.040)  [0.324] |
| **Maternal Age** |  |  | 0.016**  (0.007)  [0.021] | 0.014**  (0.007)  [0.039] | 0.014*  (0.007)  [0.060] | 0.015**  (0.007)  [0.044] | 0.016**  (0.007)  [0.029] |
| **Post-secondary Education (Mother)** |  |  |  |  | 0.034  (0.103)  [0.742] | 0.021  (0.105)  [0.843] |  |
| **R-squared** | 0.000 | 0.068 | 0.170 | 0.204 | 0.208 | 0.228 | 0.227 |
| **Adjusted R-squared** | -0.036 | 0.000 | 0.141 | 0.147 | 0.119 | 0.104 | 0.137 |
| **Model *p* value** | 0.989 | 0.386 | 0.021 | 0.041 | 0.094 | 0.152 | 0.078 |
| **No. observations** | 31 | 31 | 31 | 31 | 31 | 30 | 30 |

Standard errors are reported in parentheses, *p*-values are in brackets. *, **, *** indicate significance at the 90%, 95% and 99% level, respectively.
