# Supplementary material for: Characteristics of salivary telomere length shortening in preterm infants
Source: PLoS One. 2023 Jan 17;18(1):e0280184. doi: 10.1371/journal.pone.0280184 (PMC9844854; doi:10.1371/journal.pone.0280184)
Supplement: S5 Table — (DOCX) [file pone.0280184.s005.docx]

**Supplemental Table 5: Post-hoc power analysis**

| Dependent Variable | Independent Variable | Group 1 Mean (n) | Group 2 Mean (n) | Power* |
| --- | --- | --- | --- | --- |
| ln(TL) at birth | Maternal smoking | 2.63 (50) | 2.74 (9) | 0.30 |
|  | Sex | 2.60 (28) | 2.68 (31) | 0.29 |
|  | DEPCAT | 2.60 (33) | 2.69 (26) | 0.33 |
| TL shortening | Maternal smoking | 3.31 (41) | 4.89 (7) | 0.33 |
|  | Sex | 2.99 (24) | 4.09 (24) | 0.31 |
|  | DEPCAT | 2.98 (25) | 4.14 (23) | 0.34 |

*Reported values are from post-hoc power analyses comparing the difference between two independent means (one-tailed).
